# Aspirin use in relation to long-term survival after gastrectomy for gastric adenocarcinoma

**DOI:** 10.1007/s10120-022-01282-0

**Published:** 2022-02-15

**Authors:** Dag Holmberg, Joonas H. Kauppila, Fredrik Mattsson, Johannes Asplund, Wilhelm Leijonmarck, Shao-Hua Xie, Jesper Lagergren

**Affiliations:** 1 Upper Gastrointestinal Surgery, Department of Molecular Medicine and Surgery, Karolinska Institutet and Karolinska University Hospital, Retzius Street 13A, 4thFloor, 171 77 Stockholm, Sweden; 2 Department of Surgery, Oulu University Hospital and University of Oulu, Oulu, Finland; 3 School of Cancer and Pharmacological Sciences, King’s College London, London, UK

**Keywords:** Aspirin, Chemoprevention, Gastric neoplasm, Gastrectomy

## Abstract

**Background:**

Low-dose aspirin use may reduce cancer incidence and mortality, but its influence on gastric adenocarcinoma survival is unclear. This study aimed to assess whether aspirin use improves long-term survival following gastrectomy for gastric adenocarcinoma.

**Methods:**

This population-based cohort study included almost all patients who underwent gastrectomy for gastric adenocarcinoma in Sweden from 2006 to 2015, with follow-up throughout 2020. Preoperative exposure to a daily low-dose (75–160 mg) aspirin for 1 (main exposure), 2 and 3 years and for 1 year after gastrectomy was examined in relation to 5-year all-cause mortality (primary outcome) and disease-specific mortality. Multivariable Cox regression provided hazard ratios (HR) with 95% confidence intervals (CI), adjusted for age, sex, education, calendar year, comorbidity, statin use, tumour location, tumour stage, neoadjuvant chemotherapy, surgeon volume of gastrectomy and surgical radicality.

**Results:**

Among 2025 patients, 545 (26.9%) used aspirin at the date of gastrectomy. Aspirin use within 1 year before surgery did not decrease the adjusted risk of 5-year all-cause mortality (HR = 0.98, 95% CI 0.85–1.13) or disease-specific mortality (HR = 1.00, 95% CI 0.86–1.17). Preoperative aspirin use for 2 years (HR = 0.98, 95% CI 0.84–1.15) or 3 years (HR = 0.94, 95% CI 0.79–1.12) did not decrease the risk of 5-year all-cause mortality. Patients remaining on aspirin during the first year after gastrectomy had a similar 5-year all-cause mortality as non-users of aspirin (HR = 1.01, 95% CI 0.82–1.25).

**Conclusions:**

Low-dose aspirin use might not improve long-term survival after gastrectomy for gastric adenocarcinoma and may thus not be a target for adjuvant therapy in this group of patients.

## Introduction

Gastric cancer (> 95% adenocarcinoma) is characterized by high incidence and poor survival, making it the third most common cause of cancer deaths globally [1]. In Europe, the overall 5-year survival rate is approximately 25% [2, 3]. Surgery with total or subtotal gastrectomy, with or without pre- or perioperative chemotherapy, is the main curatively intended treatment [4].

Low-dose aspirin is commonly prescribed to prevent cardio- and cerebrovascular disease, but may also prevent cancer development and improve survival in patients with some cancer types [5, 6]. Aspirin inhibits the formation of pro-inflammatory prostaglandins, a product of the cyclooxygenase (COX)-1 and -2 complexes, which have been implicated in the formation of several neoplasias [7, 8]. Aspirin has also been suggested to counteract tumour growth and metastases in adenomatous neoplasia, possibly through blocking the production of thromboxane A2 in platelets, which counteracts platelet aggregation and may thus reduce tumour spread [9, 10]. These results have prompted the initiation of randomized clinical trials assessing aspirin as a novel adjuvant treatment to surgery in patients with certain cancer types. Regarding gastric adenocarcinoma specifically, observational studies have indicated that aspirin may reduce its incidence [11–13], but there is a paucity of data regarding a potential survival benefit of aspirin as an adjuvant therapy to gastrectomy in patients with this cancer.

This study set out to assess the hypothesis that preoperative and postoperative use of low-dose aspirin improves survival in patients who undergo gastrectomy for gastric adenocarcinoma.

## Methods

### Design

This was a nationwide Swedish population-based cohort study, titled the Swedish Gastric Cancer Surgery Study (SWEGASS), which has been presented in detail in a recent cohort description [14]. The cohort included 98% of all patients having undergone gastrectomy for gastric adenocarcinoma (including Siewert type III tumours of the gastric cardia) in Sweden from July 1, 2006 to December 31, 2015 and for the purpose of the present study, the follow-up was updated until December 31, 2020. Potentially eligible patients were initially identified in the Swedish Cancer Registry and Swedish Patient Registry by the disease, histopathology and surgery codes defining gastric adenocarcinoma and gastrectomy. The final cohort was then selected after a review of medical records from all patients, including notes from histopathology reports, multidisciplinary meetings, surgery and hospital discharge [14]. The study was approved by the Regional Ethical Review Board in Stockholm, Sweden (registration number 2017/141-31/2).

### Exposure

The main exposure was a daily dispensation of low-dose aspirin (75–160 mg) within 1 year prior to surgery. There were three secondary exposures: aspirin use for 2 years prior to surgery, 3 years prior to surgery and during the first postoperative year. The data on aspirin use came from the Swedish Prescribed Drug Registry, which electronically and automatically records all prescribed and dispensed drugs in Sweden (except for in-hospital use). The registry is nearly 100% complete [15]. Low-dose aspirin is only available by prescription in Sweden.

### Outcomes

The main outcome was 5-year all-cause mortality, defined as death from any cause occurring between the date of gastrectomy and 5 years postoperatively. The secondary outcome was 5-year disease-specific mortality, defined as death from gastric cancer as an underlying or contributing cause of death within 5 years of the gastrectomy. Information on the mortality outcomes was obtained from the Swedish Cause of Death Registry. This registry has 100% completeness for date of death and 96% completeness for cause of death in all Swedish residents and also includes deaths among Swedish residents who die abroad [16]. Because information on date of death is updated continuously, while causes of death are assembled at the end of each calendar year, follow-up for all-cause mortality was one year longer (December 31, 2020) than for disease-specific mortality (December 31, 2019).

### Covariates

We considered the following eleven covariates with categorizations in parenthesis: age (continuous), sex (male or female), education (≤ 9 years, 10–12 years or > 12 years of formal education), calendar year (continuous), comorbidity (Charlson comorbidity index score 0, 1 or ≥ 2), statin use (yes or no), tumour location (cardia or non-cardia), pathological tumour stage (0–I, II, III or IV), neoadjuvant chemotherapy (yes or no), annual surgeon volume of gastrectomy (quartiles, i.e. four equal-sized groups) and radicality of the surgical resection (R0 or R1/R2). The data on age, sex, education, calendar year, comorbidity and statin use were obtained from three nationwide complete Swedish registries: Patient Registry, Longitudinal Integrated Database for Health Insurance and Labour Market Studies (LISA) and Prescribed Drug Registry [15, 17, 18]. Comorbidity was classified based on the most well-validated version of the Charlson comorbidity index [19]. Statin use was included as a covariate since it is often used alongside aspirin and may improve gastric cancer survival [20, 21]. Information on tumour location, tumour stage, neoadjuvant chemotherapy, surgeon volume and surgical radicality was retrieved from a review of medical records.

### Statistical analysis

The study patients were followed up from the date of gastrectomy until death, 5 years after surgery or end of the study period, whichever occurred first. The cumulative survival as a function of time was estimated using the Kaplan–Meier estimator for a descriptive comparison of users and non-users of aspirin. Cox proportional hazards models were used to calculate hazard ratios (HR) with 95% confidence intervals (CI), comparing the hazard rates of mortality in aspirin users with non-users of aspirin (reference group in all analyses). A multivariable model was adjusted for the eleven covariates and categorizations presented above. In a sensitivity analysis, the study cohort was restricted to patients with curatively intended treatment. To further evaluate whether potential associations between exposure and outcome were modified by covariates, an interaction term was included in the models. HRs with 95% CI were derived within each stratum for age (≤ 66, 67–76 and ≥ 77 years), sex (male or female), comorbidity (Charlson comorbidity index score 0, 1 and ≥ 2) and tumour stage (0–I, II, III and IV). This was done for each covariate separately. The missing data were found in at least one of the eleven covariates in 12% of patients. To manage missing data, both multiple imputation and complete case analyses were conducted. In the multiple imputation analysis, the number of imputed datasets were 20 and the monotone logistic method in PROC MI was used with the assumption that missing occurred at random [22]. Imputation was conducted separately for the two outcomes and included the eleven covariates in the multivariable model. PROC MIANALYZE was used to combine the results from the analyses of the 20 datasets. Because the results from the multiple imputation analysis were considered less prone to bias and the results were similar to those of the complete case analysis, we only present the results from the multiple imputation. The proportional hazards assumption was evaluated using log–log survival plots and by calculating the correlations between Schoenfeld residuals for a particular covariate and ranking of individual failure time. The correlations were low, indicating that the proportional hazards assumption was met for all analyses. A senior biostatistician (FM) conducted the data management and statistical analyses according to a detailed and pre-defined study protocol and used the statistical software SAS/STAT Statistical Package, Version 9.4 (SAS Institute Inc., Cary, NC, USA) for these purposes.

## Results

### Patients

The study included 2025 patients who underwent gastrectomy for gastric adenocarcinoma and they contributed a total of 5684 person-years and a mean of 2.8 person-years. Of these patients, 545 (26.9%) patients used aspirin at the time of surgery. Among the aspirin users, 178 (32.7%) did not dispense any further aspirin after surgery. Some 56 (2.8%) patients started using aspirin within one year of gastrectomy. Aspirin users were generally older, more often men, had lower education, more comorbidities, more statin use, more severe complications (Clavien Dindo ≥ 3), lower rate of neoadjuvant chemotherapy and a higher rate of sub-total compared to total gastrectomy, whereas other variables were more similarly distributed (Table 1). Mortality within 90 days of surgery occurred in 52 (9.5%) of the aspirin users and 91 (6.2%) of the non-users of aspirin.

**Table 1:** Characteristics of 2,025 patients who underwent gastrectomy for gastric adenocarcinoma

	Number (%)	
	Aspirin users	Non-users of aspirin
Total	545 (100.0)	1480 (100.0)
Mean age (standard deviation)	75.4 (8.2)	67.6 (12.0)
Sex		
Men	354 (65.0)	813 (54.9)
Women	191 (35.0)	667 (45.1)
Education level (years)		
≤ 9	269 (49.4)	561 (37.9)
10–12	201 (36.9)	603 (40.7)
> 12	58 (10.6)	286 (19.3)
Not known	17 (3.1)	30 (2.0)
Calendar period		
< 2011	293 (53.8)	753 (50.9)
≥ 2011	252 (46.2)	727 (49.1)
Charlson comorbidity index		
0	119 (21.8)	742 (50.1)
1	185 (33.9)	476 (32.2)
≥ 2	241 (44.2)	262 (17.7)
Statin use		
Yes	263 (48.3)	168 (11.3)
No	282 (51.7)	1,312 (88.7)
Tumour localization		
Cardia	59 (10.8)	169 (11.4)
Non-cardia	481 (88.3)	1,304 (88.1)
Not known	5 (0.9)	7 (0.5)
Tumour stage		
0-I	149 (27.3)	337 (22.8)
II	157 (28.8)	419 (28.3)
III	182 (33.4)	529 (35.7)
IV	40 (7.3)	155 (10.5)
Not known	17 (3.1)	40 (2.7)
Histological subtype		
Diffuse	152 (27.9)	537 (36.3)
Intestinal	204 (37.4)	469 (31.7)
Mixed	17 (3.1)	61 (4.1)
Indeterminate	1 (0.2)	8 (0.5)
Not known	171 (31.4)	405 (27.4)
Neoadjuvant chemotherapy		
Yes	80 (14.7)	510 (34.5)
No	459 (84.2)	960 (64.9)
Not known	6 (1.1)	10 (0.6)
Type of gastrectomy		
Total gastrectomy	199 (36.5)	686 (46.4)
Subtotal gastrectomy	322 (59.1)	747 (50.5)
Not known	24 (4.4)	47 (3.1)
Surgeon volume		
< 2.3	146 (26.8)	360 (24.3)
2.3–3.9	146 (26.8)	358 (24.2)
4.0–5.7	133 (24.4)	365 (24.7)
> 5.7	117 (21.5)	386 (26.1)
Not known	3 (0.6)	11 (0.7)
Surgical radicality		
Yes (R0)	452 (82.9)	1,198 (80.9)
No (R1)	63 (11.6)	183 (12.4)
Not known	30 (5.5)	99 (6.7)
Postoperative complications (Clavien-Dindo)		
None	305 (56.0)	917 (62.0)
I	21 (3.9)	32 (2.2)
II	94 (17.2)	270 (18.2)
≥ III	125 (22.9)	261 (17.6)

### Aspirin use and risk of mortality

**Fig. 1:**
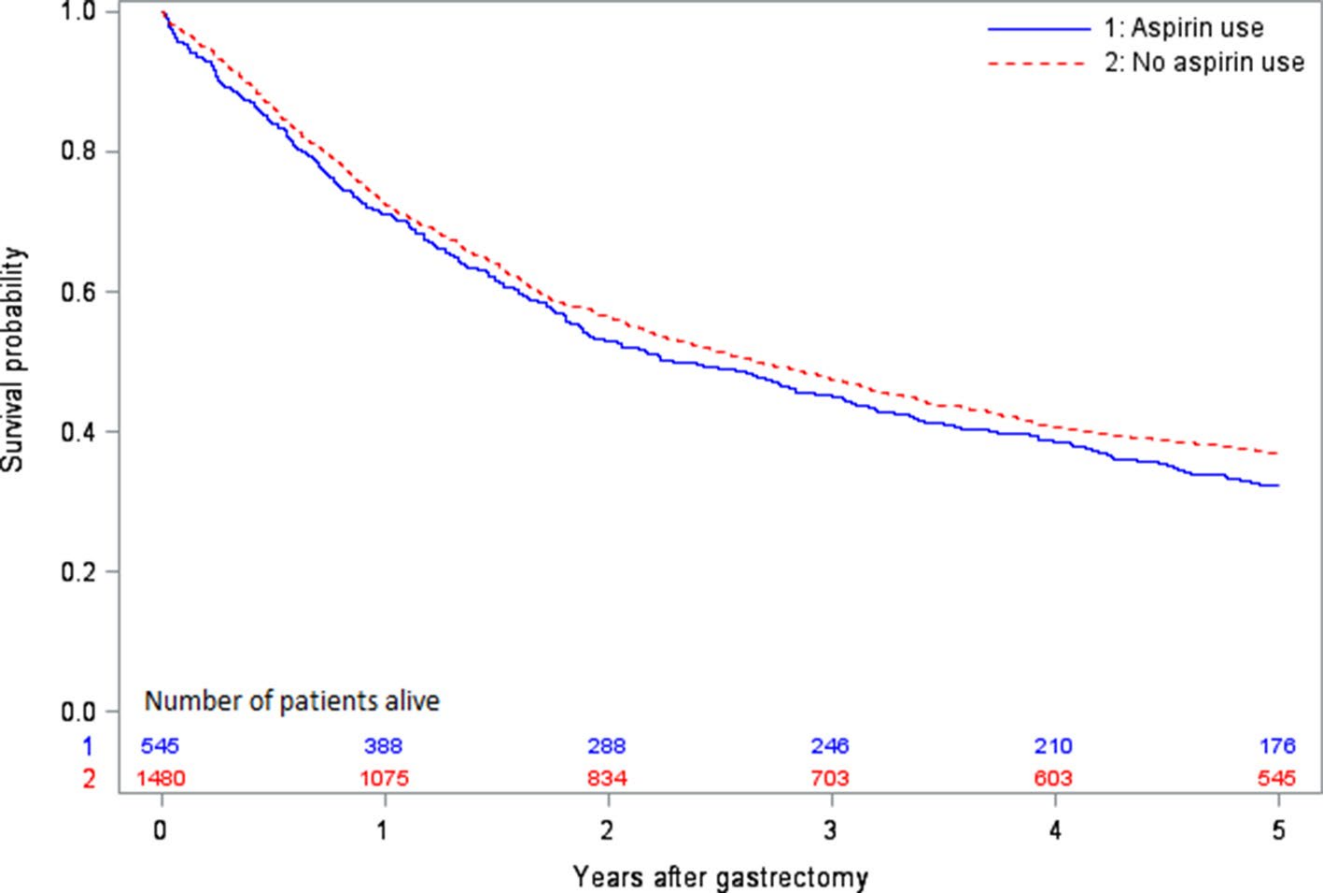
Survival probability following curatively intended gastrectomy for gastric adenocarcinoma among users of aspirin versus non-users of aspirin

The Kaplan–Meier curves were similar comparing users and non-users of aspirin (Fig. 1). Aspirin use within 1 year prior to gastrectomy was not associated with any decreased risk of 5-year all-cause mortality (adjusted HR 0.98, 95% CI 0.85–1.13) or 5-year disease-specific mortality (adjusted HR 1.00, 95% CI 0.86–1.17) (Table 2). Stratified analyses showed similar results across age groups, sexes, comorbidity scores and tumour stages (Table 2). Patients using daily aspirin for 2 and 3 years prior to surgery did not show any decreased adjusted HRs of 5-year all-cause mortality (HR 0.98, 95% CI 0.84–1.15, for 2 years and HR 0.94, 95% CI 0.79–1.12, for 3 years). Likewise, patients using aspirin both preoperatively and for 1 year postoperatively did not show any decreased risk of 5-year all-cause mortality (adjusted HR 1.01, 95% CI 0.82–1.25).

**Table 2:** Aspirin use and risk of 5-year all-cause and disease-specific mortality among 2,025 patients who underwent gastrectomy for gastric adenocarcinoma

				All-cause mortality		Disease-specific mortality	
	At risk (n)	Person-years	Deaths (n)	Unadjusted HR (95%CI)	Adjusted HR (95%CI)	Unadjusted HR (95%CI)	Adjusted HR (95%CI)
Overall							
Aspirin							
No	1,480	4,210	935	1.00 (reference)	1.00 (reference)	1.00 (reference)	1.00 (reference)
Yes	545	1,478	369	1.12 (0.99–1.26)	0.98 (0.85–1.13)	1.05 (0.92–1.20)	1.00 (0.86–1.17)
≤ 66 years							
Aspirin							
No	614	1,965	344	1.00 (reference)	1.00 (reference)	1.00 (reference)	1.00 (reference)
Yes	79	242	45	1.06 (0.77–1.44)	1.04 (0.75–1.44)	1.04 (0.75–1.45)	1.10 (0.78–1.56)
67–76 years							
Aspirin							
No	491	1,345	314	1.00 (reference)	1.00 (reference)	1.00 (reference)	1.00 (reference)
Yes	197	557	123	0.95 (0.77–1.17)	0.93 (0.75–1.17)	0.89 (0.71–1.11)	0.91 (0.82–1.16)
≥ 77 years							
Aspirin							
No	375	899	277	1.00 (reference)	1.00 (reference)	1.00 (reference)	1.00 (reference)
Yes	269	679	201	0.97 (0.81–1.16)	1.02 (0.84–1.24)	0.98 (0.80–1.20)	1.07 (0.86–1.33)
Men							
Aspirin							
No	813	2,290	515	1.00 (reference)	1.00 (reference)	1.00 (reference)	1.00 (reference)
Yes	354	970	241	1.09 (0.94–1.27)	0.92 (0.77–1.09)	1.01 (0.85–1.19)	0.90 (0.74–1.09)
Women							
Aspirin							
No	667	1,920	420	1.00 (reference)	1.00 (reference)	1.00 (reference)	1.00 (reference)
Yes	191	509	128	1.15 (0.94–1.40)	1.09 (0.88–1.35)	1.14 (0.92–1.40)	1.19 (0.95–1.49)
No comorbidity							
Aspirin							
No	742	2,321	424	1.00 (reference)	1.00 (reference)	1.00 (reference)	1.00 (reference)
Yes	119	351	73	1.14 (0.89–1.46)	1.12 (0.87–1.45)	1.05 (0.80–1.37)	1.11 (0.84–1.47)
1 comorbidity							
Aspirin							
No	476	1,356	305	1.00 (reference)	1.00 (reference)	1.00 (reference)	1.00 (reference)
Yes	185	532	115	0.97 (0.78–1.20)	1.02 (0.81–1.28)	0.97 (0.78–1.22)	1.08 (0.85–1.38)
≥ 2 comorbidities							
Aspirin							
No	262	533	206	1.00 (reference)	1.00 (reference)	1.00 (reference)	1.00 (reference)
Yes	241	596	181	0.81 (0.66–0.98)	0.87 (0.70–1.07)	0.77 (0.62–0.97)	0.87 (0.69–1.11)
Tumour stage 0-I							
Aspirin							
No	337	1,457	80	1.00 (reference)	1.00 (reference)	1.00 (reference)	1.00 (reference)
Yes	149	557	62	1.92 (1.38–2.67)	1.46 (1.04–2.05)	1.78 (1.14–2.78)	1.45 (0.92–2.28)
Tumour stage II							
Aspirin							
No	419	1,384	240	1.00 (reference)	1.00 (reference)	1.00 (reference)	1.00 (reference)
Yes	157	517	98	1.08 (0.86–1.36)	0.80 (0.63–1.03)	1.05 (0.82–1.36)	0.85 (0.64–1.11)
Tumour stage III							
Aspirin							
No	529	1,052	444	1.00 (reference)	1.00 (reference)	1.00 (reference)	1.00 (reference)
Yes	182	316	158	1.17 (0.98–1.40)	0.88 (0.72–1.07)	1.15 (0.96–1.39)	0.93 (0.75–1.15)
Tumour stage IV							
Aspirin							
No	155	234	139	1.00 (reference)	1.00 (reference)	1.00 (reference)	1.00 (reference)
Yes	40	37	39	1.64 (1.15–2.34)	1.47 (1.01–2.13)	1.59 (1.10–2.30)	1.46 (0.99–2.14)

^*^Adjusted for age, sex, education, calendar year, comorbidity, statin use, tumour location, tumour stage, neoadjuvant chemotherapy, surgeon volume and surgical radicality

### Sensitivity analysis

In a sensitivity analysis, we excluded 291 (14.3%) patients who underwent gastrectomy without a clearly curative intent. In this analysis of 1,734 patients, the 90-day mortality rate was 4.0%. Aspirin use was not associated with either 5-year all-cause mortality (adjusted HR 0.92, 95% CI 0.78–1.09) or 5-year disease-specific mortality (adjusted HR 0.94, 95% CI 0.79–1.13), compared to non-users.

## Discussion

This study found no support for the hypothesis that aspirin use improves the 5-year survival in patients who undergo gastrectomy for gastric adenocarcinoma. These null results were consistent across ages, sexes, comorbidity scores, tumour stages, lengths of preoperative use of aspirin and in patients who continued using aspirin after gastrectomy, as well as in patients who underwent clearly curatively intended surgery.

Methodological strengths of this study include the population-based design with full national coverage, complete and long-term follow-up of all patients, large cohort size, prospectively collected and detailed clinical data with low proportions of missing and adjustment for all established and several potential prognostic factors. The information on the exposures (aspirin use), outcomes (5-year mortality) and covariates was accurate. Only adenocarcinoma was included because other histological types of gastric malignancies may have different treatment and survival. There are also weaknesses. Low-dose aspirin requires a prescription in Sweden, but small packages of high-dose aspirin are available over-the-counter, which might introduce limited misclassification of the exposure. The observational design is subject to unknown or residual confounding. Some exposures that could have confounded the findings are tobacco smoking, alcohol overconsumption and obesity. However, although these exposures were not directly adjusted for, they were still accounted for to some extent by the adjustments for comorbidities related to these exposures. Residual confounding by comorbidity related to aspirin use is also unlikely given that the null results observed for the all-cause mortality outcomes were confirmed in the analyses of gastric adenocarcinoma-specific mortality. Data on chemotherapy-related fever were not available, but were unlikely to interact with aspirin use given that continuous use was required and low-dose aspirin is not prescribed as an anti-pyretic agent.

The role of low-dose aspirin as a potential anticarcinogenic agent has been studied intensively during the last decades. This research has been encouraged by the results of a long-term follow-up of five randomized clinical trials with 17,285 participants who randomly received aspirin or placebo/control medication for the prevention of cardiovascular disease [10]. In that study, aspirin users were at a 31% decreased relative risk of metastasis upon diagnosis of various adenocarcinoma sites (including the stomach) and among aspirin users with a localized tumour, the risk of subsequent metastasis was 55% decreased [10]. However, the trial was not powered to examine gastric cancer separately. The evidence of a cancer-preventive effect of aspirin use has been strongest for colorectal cancer [23–25]. However, a recent randomized clinical trial showed a similar risk of mortality in users of celecoxib (a COX-2 inhibitor) and placebo in 2,524 patients with stage III colorectal cancer (HR 0.86, 95% CI 0.72–1.04) [26].

Regarding gastric cancer specifically, a meta-analysis of 33 observational studies and 1,927,971 patients indicated that aspirin use slightly decreases the risk of developing this tumour, with pooled risk ratios in fixed- and random-effects models of 0.89 (95% CI 0.87–0.91) and 0.83 (0.74–0.92), respectively [13]. Yet, only one previous study has examined whether aspirin use improves survival in patients with established gastric cancer. That was a study from the United Kingdom analysing two cohorts with a total of 1,720 patients who underwent surgery for gastric cancer and similar to the present study, aspirin use was not associated with any decreased mortality [27]. However, that study was confounded by more than 80% missing tumour stage, the most important prognostic factor of gastric cancer and included all histologies and also non-surgical patients. An ongoing trial is investigating whether aspirin use decreases mortality in gastric and oesophageal cancer, but preliminary results are not expected until 2027 [28]. In the meantime, the findings from the current study together with the previous study provide the best available evidence and may prompt an interim analysis of the trial.

In conclusion, this population-based cohort study with complete follow-up and adjustment for all known prognostic factors indicates that the use of aspirin does not decrease the all-cause or 5-year disease-specific mortality in patients who undergo curatively intended gastrectomy for gastric adenocarcinoma.

## Data Availability

The data that support the findings of this study are available from The Swedish Board of Health and Welfare. Restrictions apply to the availability of these data, which were used under license for this study. The data are available from the authors with the permission of The Swedish Board of Health and Welfare.
